# Editors' Note

**DOI:** 10.5195/ijt.2023.6600

**Published:** 2023-12-12

**Authors:** Ellen R. Cohn, Jana Cason

## Journal Overview

The International Journal of Telerehabilitation (IJT) is a biannual journal dedicated to advancing telerehabilitation by disseminating peer-reviewed information about current research and practices. IJT is indexed by PubMed and Scopus. IJT is published via the open journal system (OJS) and sponsored by the University of Pittsburgh's Office of Scholarly Communication and Publishing at the University Library System. This institutional support enables IJT to be subscription-free and require no author fees.

## International Readership and Authors

IJT enjoys a diverse international audience due to the expanding global relevance of telemedicine, telehealth, and telerehabilitation. In this Fall 2023 issue, we are pleased to publish work by authors from Australia, Canada, India, Saudi Arabia, Thailand, Ukraine, and the United States, as well as content related to Antigua and Barbados.

